# Laparoscopic surgical challenge for T4a colon cancer

**DOI:** 10.1002/ags3.12009

**Published:** 2017-04-25

**Authors:** Seishi Hojo, Hidejiro Kawahara, Masaichi Ogawa, Katsuhito Suwa, Ken Eto, Katsuhiko Yanaga

**Affiliations:** ^1^ Department of Surgery The Jikei University School of Medicine Tokyo Japan

**Keywords:** colon cancer, laparoscopic surgery, outcome, peritoneal dissemination, serosal invasion

## Abstract

For patients with T4a colon cancer, the risk of peritoneal dissemination after surgery remains unclear. Seven hundred and eleven patients with T3 or T4a colon cancer, 80 years of age or younger, underwent curative resection (open surgery in 512 and laparoscopic surgery in 199) at the four Jikei University hospitals between 2006 and 2012. Their risk factors for peritoneal dissemination after surgery were evaluated retrospectively. Number of lymph node metastases, postoperative liver metastases and postoperative peritoneal dissemination events in the T4a group were significantly greater than the number in the T3 group. Peritoneal dissemination after surgery developed in four patients (0.7%) in the T3 group and in six patients (5%) in the T4a group. Risk factors for peritoneal dissemination consisted of macroscopic type (*P* = 0.016), serosal invasion (*P* = 0.017) and number of lymph node metastases (*P* = 0.009) according to the Cox proportional hazards regression model. However, tumor diameter and surgical approach (laparoscopic *vs* open) were not significant factors for peritoneal dissemination. There were no significant differences between the postoperative relapse‐free survival rates for each surgical approach within the T3 or T4a group. Because of comparable postoperative peritoneal dissemination in T3 and T4a colon cancer by the surgical approach (laparoscopic or open), laparoscopic surgery for patients with T4a colon cancer seems justified.

## 1 Introduction

In the early 1990s, laparoscopic surgery for early‐stage cancer was considered feasible in Japan, but it was not known whether an adequate extent of lymph node dissection for more advanced cases could be achieved by laparoscopic procedures.^1^ In the Japanese Society for Cancer of the Colon and Rectum Guidelines 2010,^2^ laparoscopic surgery is suitable for D2, D1 or D0 resection of colon and RS cancer and is strongly indicated for the treatment of cStage 0 to cStage I disease. However, according to the national survey conducted by the Japanese Society of Endoscopic Surgery (JSES),^3^ the percentage of more advanced cancers (T2 or higher) accounting for the procedure has increased to over 50% of the total cases. Although many patients with T4 colon cancer are included in those cases, the risk of peritoneal dissemination after surgery remains unclear. The aim of this retrospective study was to evaluate the validity of laparoscopic surgery for patients with T4a colon cancer.

## 2 Methods

Seven hundred and eleven patients with T3 or T4a colon cancer, aged 80 years or younger, underwent curative resection (open surgery in 512 and laparoscopic surgery in 199) at the four Jikei University hospitals between 2006 and 2012, and their risk factors for peritoneal dissemination after surgery were evaluated retrospectively. The medical records of all patients were reviewed and classified according to the *Japanese Classification of Colorectal Carcinoma*.^4^ According to this classification, T3 corresponds to invasion of the subserosa and T4a to serosal invasion, excluding direct extension into adjacent structures or organs, which is classified as T4b.

No special procedure to cover serosal invasion to prevent detachment of cancer cells to the peritoneal cavity was added during surgery in patients with T4a colon cancer. Choice of surgical procedure, laparoscopic surgery or open surgery, was based on the preference of the operators. However, laparoscopic surgery was aggressively chosen in cases in which expert laparoscopic surgeons authorized by JSES were operators or assistants.

### 2.1 Follow up after surgery and postoperative adjuvant chemotherapy

All patients were followed for 5 years with measurement of serum carcinoembryonic antigen every 3 months, computed tomography (CT) every 6 months and colonoscopy every 12 months. When we suspected any recurrence, CT and positron emission tomography were carried out at that time.

For 6 months after surgery, patients with stage III disease received oral S‐1 (Taiho Pharmaceuticals Co. Ltd, Tokyo, Japan) or capecitabine (Xeloda; Hoffmann‐La Roche, Basel, Switzerland), whereas patients with stage II disease received no adjuvant chemotherapy.

### 2.2 Statistical analysis

Continuous variables were expressed as mean and range. Wilcoxon rank‐sum test was used for comparison of continuous variables and the chi‐squared test was used for comparison of categorical data. Postoperative relapse‐free survival rates were examined by the Kaplan–Meier method and log–rank analysis. Variables affecting peritoneal dissemination after surgery were analyzed using the Cox proportional hazards regression. A *P*‐value of less than.05 was considered to indicate significance. All data were analyzed with the computer program IBM SPSS Statistics, version 22.0 (IBM Japan, Ltd, Tokyo, Japan).

## 3 Results

### 3.1 Comparison of patient characteristics between T3 and T4a

Between patients with T4a disease and patients with T3 disease, no significant difference was identified in age, gender, tumor location, macroscopic type of tumor, tumor diameter, and pathological type (Table 1). The groups of patients did differ significantly in surgical approach, operation time, intraoperative bleeding, lymph node metastasis, and postoperative recurrence rates of peritoneal dissemination and liver metastasis (Table 1). Median follow‐up period was 78 months (range 36–130 months). Frequency of peritoneal dissemination after surgery was less than one percent for patients with T3 and five percent for those with T4a (Table 1).

**Table 1:** Clinicopathological characteristics of patients with T3 and T4a colon cancer

Variable	T3 (*n* = 589)	T4a (*n* = 122)	*P*‐value
Age (years)	66.0 (25–80)	66.3 (32–80)	0.565
Gender			
Male	334 (57)	77 (63)	0.192
Female	255 (43)	45 (37)	
Tumor location			
Cecum	65 (11)	16 (13)	0.116
Ascending colon	170 (29)	30 (25)	
Transverse colon	88 (15)	29 (24)	
Descending colon	59 (10)	8 (6)	
Sigmoid colon	207 (35)	39 (32)	
Surgical approach			
Open surgery	412 (70)	100 (82)	0.007
Laparoscopic surgery	177 (30)	22 (18)	
Operation time (min)	185.0 (45–595)	170.5 (65–487)	0.036
Intraoperative blood loss (mL)	167.2 (0–3210)	248.6 (0–3348)	0.011
Macroscopic type			
I	25 (4)	2 (2)	0.246
II	542 (92)	113 (93)	
III	22 (4)	7 (5)	
Tumor diameter (mm)	47.7 (13–210)	51.3 (20–170)	0.112
Pathological type			
Well‐differentiated adenocarcinoma	173 (29)	41 (33)	0.100
Moderately differentiated adenocarcinoma	383 (65)	73 (60)	
Poorly differentiated adenocarcinoma	15 (3)	7 (6)	
Others	18 (3)	1 (1)	
Stage			
II	337 (57)	43 (35)	0.001>
III	252 (43)	79 (65)	
Recurrence after surgery	55 (9)	34 (28)	0.001>
Recurrent site			
Peritoneum	4 (1>)	6 (5)	0.001>
Liver	30 (5)	20 (16)	0.001>
Lung	14 (2)	6 (5)	0.122
Others	7 (1)	2 (2)	0.685

Data are presented as mean (range) or as *n* (%).

### 3.2 Comparison of patient postoperative relapse‐free survival rate between T3 and T4a

**Figure 1:**
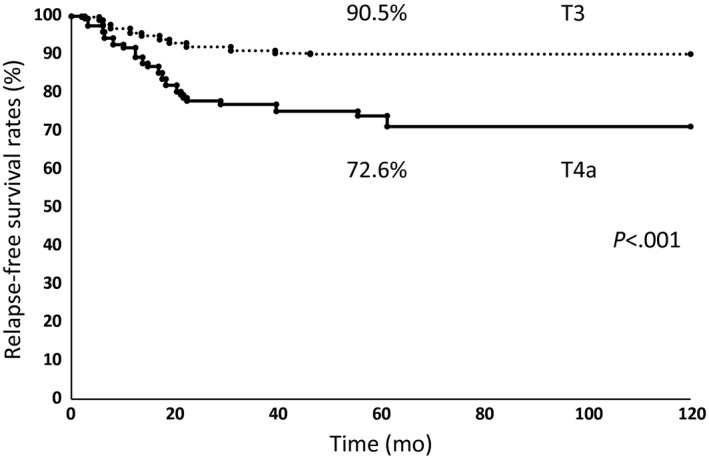
Kaplan–Meier relapse‐free survival curves for patients with T4a and T3 colon cancer

The 5‐year relapse‐free survival rates were 90.5% for patients with T3 and 72.6% for patients with T4a (Fig. 1). There was a significant difference in postoperative relapse‐free survival rates between T3 and T4a according to log–rank analysis (*P* < 0.001).

### 3.3 Comparison between open and laparoscopic surgery in patients with T3

Between patients with T3 who received open surgery and laparoscopic surgery, no significant differences were identified in age, gender, macroscopic type of tumor, pathological type, lymph node metastasis and postoperative recurrence rates or sites, whereas significant differences were achieved in tumor location, operation time, intraoperative bleeding, tumor diameter, and pathological type (Table 2). Tumor diameters in the open surgery group were significantly larger than in the laparoscopic surgery group.

**Table 2:** Comparison between open and laparoscopic surgery in patients with T3 colon cancer

Variable	Open surgery (*n* = 412)	Laparoscopic surgery (*n* = 177)	*P*‐value
Age (years)	67.9 (32–80)	64.9 (25–80)	0.001
Gender			
Male	235 (57)	99 (56)	0.804
Female	177 (43)	78 (44)	
Tumor location			
Cecum	37 (9)	28 (16)	0.001>
Ascending colon	132 (32)	40 (23)	
Transverse colon	70 (17)	16 (9)	
Descending colon	41 (10)	16 (9)	
Sigmoid colon	132 (32)	77 (43)	
Operation time (min)	173.1 (45–415)	212.8 (85–595)	0.001>
Intraoperative blood loss (mL)	214.2 (0–1950)	58.2 (0–3210)	0.001>
Macroscopic type			
I	15 (4)	10 (6)	0.525
II	381 (92)	161 (91)	
III	16 (4)	6 (3)	
Tumor diameter (mm)	51.9 (13–210)	37.4 (10–120)	0.001>
Pathological type			
Well‐differentiated adenocarcinoma	128 (31)	45 (25)	0.031
Moderately differentiated adenocarcinoma	255 (62)	128 (72)	
Poorly differentiated adenocarcinoma	14 (3)	1 (1)	
Others	15 (4)	3 (2)	
Stage			
II	227 (55)	110 (62)	0.113
III	185 (45)	67 (38)	
Recurrence after surgery	42 (10)	13 (7)	0.276
Recurrent site			
Peritoneum	2 (1>)	2 (1>)	0.744
Liver	23 (6)	7 (4)	0.410
Lung	13 (3)	1 (1>)	0.068
Others	4 (1)	3 (2)	0.742

Data are presented as mean (range) or as *n* (%).

### 3.4 Comparison of patient postoperative relapse‐free survival rate in T3 between open and laparoscopic surgery

**Figure 2:**
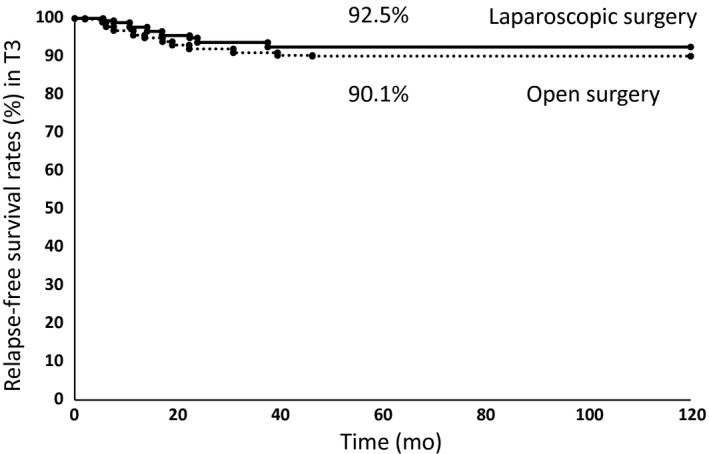
Kaplan–Meier relapse‐free survival curves for patients with T3 colon cancer who received laparoscopic surgery or open surgery

The 5‐year relapse‐free survival rates of patients with T3 were 92.5% for patients after laparoscopic surgery and 90.1% for patients after open surgery (Fig. 2). There was no significant difference in postoperative relapse‐free survival rate between the two groups by log–rank analysis (*P *= 0.338).

### 3.5 Comparison between open and laparoscopic surgery in patients with T4a

Between patients with T4 who received open surgery and patients who received laparoscopic surgery, no significant differences were found in age, tumor location, operation time, macroscopic type of tumor, tumor diameter, pathological type, lymph node metastasis and postoperative recurrence rate or site (Table 3), whereas significant differences were identified in gender and intraoperative bleeding (Table 3). Intraoperative blood loss in the open surgery group was significantly greater than in the laparoscopic surgery group.

**Table 3:** Comparison between open and laparoscopic surgery in patients with T4a colon cancer

Variable	Open surgery (*n* = 100)	Laparoscopic surgery (*n* = 22)	*P*‐value
Age (years)	67.2 (32–80)	62.5 (40–77)	0.062
Gender			
Male	68 (68)	9 (41)	0.017
Female	32 (32)	13 (59)	
Tumor location			
Cecum	12 (12)	4 (18)	0.426
Ascending colon	23 (23)	7 (32)	
Transverse colon	27 (27)	2 (9)	
Descending colon	7 (7)	1 (5)	
Sigmoid colon	31 (31)	8 (36)	
Operation time (min)	166.8 (65–487)	187.6 (110–280)	0.112
Intraoperative blood loss (mL)	298.0 (0–3348)	24.6 (0–240)	0.013
Macroscopic type			
I	2 (2)	0 (0)	0.343
II	91 (91)	22 (100)	
III	7 (7)	0 (0)	
Tumor diameter (mm)	52.3 (20–187)	46.6 (21–125)	0.410
Pathological type			
Well‐differentiated adenocarcinoma	31 (31)	10 (45)	0.391
Moderately differentiated adenocarcinoma	61 (61)	12 (55)	
Poorly differentiated adenocarcinoma	7 (7)	0 (0)	
Others	1 (1)	0 (0)	
Stage			
II	36 (36)	7 (32)	0.710
III	64 (64)	15 (68)	
Recurrence after surgery	30 (30)	4 (18)	0.392
Recurrent site			
Peritoneum	6 (6)	0 (0)	0.526
Liver	16 (16)	4 (18)	1.000
Lung	6 (6)	0 (0)	0.526
Others	2 (2)	0 (0)	1.000

Data are presented as mean (range) or as *n* (%).

### 3.6 Comparison of postoperative relapse‐free survival rate of patients with T4a between open and laparoscopic surgery

**Figure 3:**
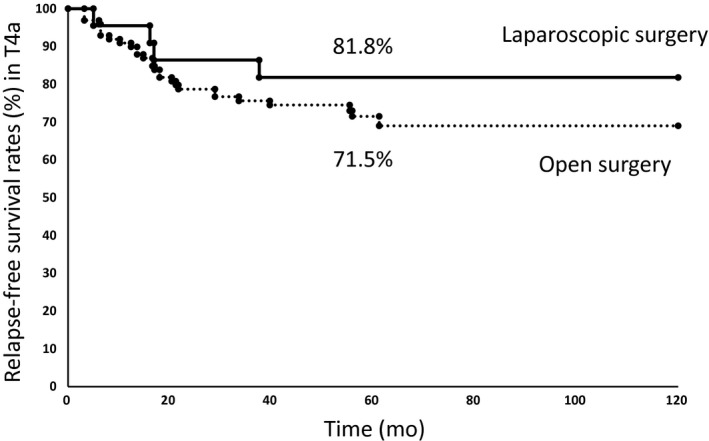
Kaplan–Meier relapse‐free survival curves for patients with T4a colon cancer who received laparoscopic surgery or open surgery

The 5‐year relapse‐free survival rate of patients with T4a was 81.8% for patients who underwent laparoscopic surgery and 71.5% for patients who underwent open surgery (Fig. 3), showing no significant difference by log–rank analysis (*P *= 0.389).

### 3.7 Cox proportional hazards regression for peritoneal dissemination after surgery

To determine the variables affecting peritoneal dissemination after surgery, 11 variables (age, gender, tumor location, operative time, intraoperative blood loss, macroscopic type, tumor diameter, pathological type, serosal invasion, number of lymph node metastases, and surgical approach) were analyzed using the Cox proportional hazards regression, because the stage identifies depth of tumor and number of lymph node metastases. Only three factors, macroscopic type (*P *= 0.016), serosal invasion (*P *= 0.017) and number of lymph node metastases (*P *= 0.009), were independent contributing factors to peritoneal dissemination after surgery (Table 4).

**Table 4:** Multivariate analyses using Cox proportional hazards regression model for peritoneal dissemination after surgery

Variable	Hazard ratio (95% confidence interval)	*P*‐value
Age (years)		
≦70	2.399 (0.584–9.865)	0.225
>70	1	
Gender		
Male	1.085 (0.279–4.209)	0.906
Female	1	
Tumor location		
Left colon	0.999 (0.479–2086)	0.999
Right colon	1	
Operation time (min)		
≦180	0.726 (0.163–3.231)	0.674
>180	1	
Intraoperative blood loss (mL)		
≦300	1.188 (0.264–5.346)	0.822
>300	1	
Macroscopic type		
Others	7.359 (1.454–37.248)	0.016
Type II	1	
Tumor diameter (mm)		
≦50	0.953 (0.253–3.586)	0.943
>50	1	
Pathological type		
Well‐ and moderately differentiated carcinoma	0.744 (0.251–2.206)	0.593
Others	1	
Serosal invasion		
Positive	5.174 (1.347–19.871)	0.017
Negative	1	
No. lymph node metastases		
≦4	7.399 (1.646–33.269)	0.009
>4	1	
Surgical approach		
Laparoscopic surgery	1.515 (0.265–8.649)	0.640
Open surgery	1	

## 4 Discussion

Although liver metastasis is the most frequent recurrence pattern after surgery in patients with colon cancer, peritoneal dissemination accounted for 16% of all patients with recurrence, for which serosal invasion may correlate with peritoneal dissemination.^5^ The recent frequency of peritoneal dissemination in T4 colon cancer after surgery is unknown.^6^ Nishikawa *et al*.^7^ reported that 14% of 151 patients with T4 colorectal cancer had positive cytology detected by peritoneal lavage cytology during surgery, and 64.5% of patients with T3 or T4 colorectal cancer with positive peritoneal lavage cytology developed peritoneal dissemination. However, patients with positive cytology who did not develop peritoneal dissemination during the operation could achieve long‐term survival.

In our study, peritoneal dissemination after surgery developed in six patients with T4a (5%), which was very low compared to the previous reports evaluated more than 10 years ago.^5^, ^7^ Cox proportional hazards regression analysis demonstrated serosal invasion and number of lymph node metastases to be the independent contributing factors for peritoneal dissemination after surgery, whereas the surgical approach failed to demonstrate a significant difference in the postoperative relapse‐free survival rate in the T4a group. Whether we chose laparoscopic or open surgery for the T4a group, the surgical outcome was the same. Therefore, laparoscopic surgery for patients with T4a colon cancer seems justified.

A large number of controlled studies and meta‐analyses have shown that laparoscopic surgery is associated with less pain, early recovery of bowel transit and shorter hospital stay compared to open surgery.^8^, ^9^, ^10^, ^11^, ^12^ Furthermore, a subset analysis of a randomized trial showed a lower recurrence rate and better survival in patients with stage III colon cancer undergoing laparoscopic surgery compared with open surgery.^13^, ^14^, ^15^, ^16^, ^17^ In those studies, no additional procedure to cover serosal invasion to prevent the detachment of cancer cells to the peritoneal cavity was used during surgery in either approach.

In conclusion, laparoscopic surgery for patients with T4a colon cancer seems justified because patients with T3 and T4a had comparable postoperative peritoneal dissemination and other recurrences such as liver or lung metastasis.
